# Analgesic Effect Comparison Between Nalbuphine and Sufentanil for Patient-Controlled Intravenous Analgesia After Cesarean Section

**DOI:** 10.3389/fphar.2020.574493

**Published:** 2020-11-16

**Authors:** Shen Sun, Yundong Guo, Tingting Wang, Shaoqiang Huang

**Affiliations:** Department of Anesthesiology, Obstetrics and Gynecology Hospital of Fudan University, Shanghai, China

**Keywords:** cesarean section, post-operative pain, nalbuphine, sufentanil, analgesic

## Abstract

**Background**: Efficient maternal pain relief after cesarean delivery remains challenging, but it is important to improve outcomes for the mother and the newborn during the puerperium. We compared the analgesic effect of nalbuphine (a κ receptor agonist/μ receptor antagonistic) with that of sufentanil (a *µ*-receptor agonist) in patient-controlled intravenous analgesia (PCIA) after cesarean section.

**Methods**: We enrolled 84 patients scheduled for elective cesarean sections with spinal anesthesia and randomized them into either nalbuphine or sufentanil groups (42 patients each). Pain scores, PCIA drug consumptions, degree of satisfaction, and adverse events were recorded as outcome measures.

**Results**: The pain scores at rest and uterine cramping pain scores in the nalbuphine group were lower than those in the sufentanil group at 6, 12, and 24 h after the operation. Also, the pain scores while switching to a seated position were lower in the nalbuphine group than in the sufentanil group at 6 and 12 h after the operation (*p* < 0.05). We found no significant differences in the PCIA drug consumption between the two groups. The degree of satisfaction in patients in the nalbuphine group was higher than that of patients in the sufentanil group (*p* = 0.01). Adverse events did not differ in the two groups.

**Conclusion**: PCIA with nalbuphine provides better analgesia and higher patient satisfaction than sufentanil after cesarean section.

## Introduction

It is well-known that surgical procedures are a common cause of acute pain (Robert et al., 2015), and cesarean section is one the most common inpatient surgery performed worldwide (Eisenach et al., 2008). Owing to significant trauma of the internal organs caused by the cesarean section, effective analgesia in the post-operative period can be challenging. Furthermore, the uterine contraction agent used after the procedure to promote uterine involution and reduce postoperative hemorrhages can supplement the noxious stimuli and cause cramping pain (Lavand’homme, 2006).

Inadequate pain control can have several adverse effects in patients undergoing surgery. Acute postoperative pain is considered a risk factor for chronic pain (Callesen et al., 1999; Perkins and Kehlet, 2000) and may also lead to increased morbidity and prolonged hospital stay. For patients undergoing cesarean sections, ineffective pain control can hamper breastfeeding and care of the newborn. Several different regimens have been used for post-cesarean analgesia, however, it is estimated that more than 20% of patients still experience severe postoperative pain (Eisenach et al., 2008).

Despite several clinical trials evaluating different analgesic modalities, no clear guidelines exist for the management of pain after cesarean section (Sutton and Carvalho, 2017). Multimodal analgesia is usually recommended for all patients that include interventions like neuraxial opioids under regional anesthesia or transversus abdominis plane blocks in patients under general anesthesia. These modalities are often combined with post-operative intravenous or oral opioids/non-steroid anti-inflammatory drugs (NSAIDs) to prolong the duration of analgesia (Sutton and Carvalho, 2017). An alternate modality to overcome the short duration of action of neuraxial analgesia is by patient-controlled epidural catheters (PCEA). PCEA is known to provide optimal pain relief after cesarean sections (Schenk et al., 2006; Zhu et al., 2013). However, slippage or dislocation of the epidural catheter and risk of infection limit the clinical application of PCEA (Paech et al., 1994; Ngan Kee et al., 1997; Halpern et al., 2004; Schenk et al., 2006; Zhu et al., 2013). In comparison to PCEA, Patient-controlled intravenous analgesia (PCIA) is an effective method of pain control after surgery with minimal complications. PCIA has shown to reduce drug consumption, improve patient satisfaction, with shorter hospital stays and fewer adverse effects (Chi et al., 2017). Enhancing the effects of PCIA may significantly impact the recovery of patients after the cesarean section (Saracoglu et al., 2012).

Sufentanil is a highly potent opioid commonly used for PCIA (Nie et al., 2014; Kaufner et al., 2016). This *µ*-receptor agonist is known to provide better analgesia and reduced respiratory depression as compared to an equivalent dose of fentanyl. The drug has a rapid peak and short half-life which makes it ideal for PCIA (Scott et al., 1991). However, sufentanil like other opioids may induce adverse reactions like nausea, vomiting, pruritis, dizziness, drowsiness, constipation, urinary retention, and respiratory depression (Gadsden et al., 2005).

Nalbuphine is a κ receptor agonist and *µ* receptor antagonistic with a duration of action of approximately 3–6 h. Specific κ receptor agonist and gene knockout experiments have revealed that κ receptor agonists block visceral pain induced by chemical stimulation with better efficacy as compared to pure *µ* opioid receptor agonist (Pasternak, 2005). It has been shown that the use of nalbuphine carries a lower risk of adverse events like nausea, vomiting, pruritus, constipation, and respiratory depression. (Jaillon et al., 1989). Thus, nalbuphine may be a suitable alternative to sufentanil with a better safety profile. Recently, Xi et al. (2020) in a double-blind randomized controlled trial have demonstrated that nalbuphine offers better postoperative analgesia as compared to sufentanil in orthognathic surgery patients. Furthermore, studies on patients undergoing colonoscopy and total hysterectomy have also found nalbuphine to be a suitable alternative to sufentanil (Deng et al., 2017; Sun et al., 2020). While intrathecal nalbuphine has been used for postoperative analgesia after cesarean sections (Culebras et al., 2000), its use for PCIA has received limited attention. Moreover, to the best of our knowledge, no study has compared nalbuphine with sufentanil in cesarean section patients. Therefore, this prospective randomized study was designed to compare the analgesic effects of nalbuphine with sufentanil for PCIA after cesarean section.

## Materials and Methods

### Inclusion Criteria

This study was conducted at the Department of Anesthesiology, Obstetrics and Gynecology Hospital of Fudan University, Shanghai, China. Ethical clearance was obtained from the institutional ethical committee before the conduct of the trial (approval no: 2017-22). The study protocol was pre-registered on https://clinicaltrials.gov/ with registration no NCT02604797. Primipara patients scheduled for elective lower segment cesarean section with spinal anesthesia between January 2017 and May 2017 were enrolled in the trial. Patients within the age range of 20–40 years and height between 155–175 cm were included. Patients with pregnancies complicated with hypertension, severe preeclampsia, diabetes mellitus, cardiac or renal disease; those with gestation age <37 weeks; with a long history of NSAIDs or opioid analgesic use; and those with chronic pain disorders were excluded. Informed written consent was obtained from all included patients.

### Randomization

Patients were randomized into two groups, stratified based on age and body weight, using two sets of randomized medication codes generated by the computer. The sealed codes were preserved by the medication staff and follow-up manager. The women in the nalbuphine group received nalbuphine (100 mg) and ramosetron (0.3 mg) while those in the sufentanil group received sufentanil (100 *μ*g) and ramosetron (0.3 mg). Both groups of patients were managed with a continuous dose of 1 ml h^−1^ and a bolus dose of 1 ml, with a lock-out of 10 min, and a maximum PCIA dose per hour of 10 ml. The number of bolus doses per patient was controlled by the maximum PCIA dose per hour and the lock-out time. The test drugs diluted in 10 ml of saline were randomly selected by the computer half an hour before the intervention. A researcher handed the drug over to the operator for connecting it to the analgesic pump at the end of the operation. This researcher was not involved in any other part of the trial or the data collection process.

### Procedure

None of the patients were premedicated. The temperature of the operation room was maintained at 22°C. An anesthesiologist commenced monitoring including electrocardiography, heart rate (HR), systolic blood pressure (SBP), diastolic blood pressure (DBP), mean arterial pressure, and pulse oxygen saturation (SpO_2_) using an S/5 Anesthesia Monitor (GE, Finland). A 500 ml hydroxyethyl starch solution was infused at 0.2 ml kg^−1^·min^−1^ followed by Lactated Ringer's solution at the same rate till the end of every operation. A combined spinal-epidural anesthesia procedure was performed at the L_3-4_ intervertebral space. Briefly, 10 mg (2 ml) of isobaric bupivacaine 0.5% was diluted into 2.5 ml with cerebrospinal fluid and injected into the subarachnoid space over 15–30 s. The sensory block level to cold was inspected every 3 min (1, 4, 7, and 10 min) with alcohol swabs, and was recorded at the time point of 10 min. Hypotension was defined as SBP lower than 80% of the baseline value and was managed with intravenous phenylephrine 100 *µ*g as necessary. Severe sinus bradycardia (HR <50 beats/min) was treated with intravenous administration of 0.3 mg atropine. The surgery commenced when the sensory block reached the T6 level. Patients unable to achieve this level were excluded from the study. The anesthesiologist provided 1.5% lidocaine through the epidural catheter for women with inadequate sensory block. During the operation, a uterine contraction agent was administered as needed.

**FIGURE 1:**
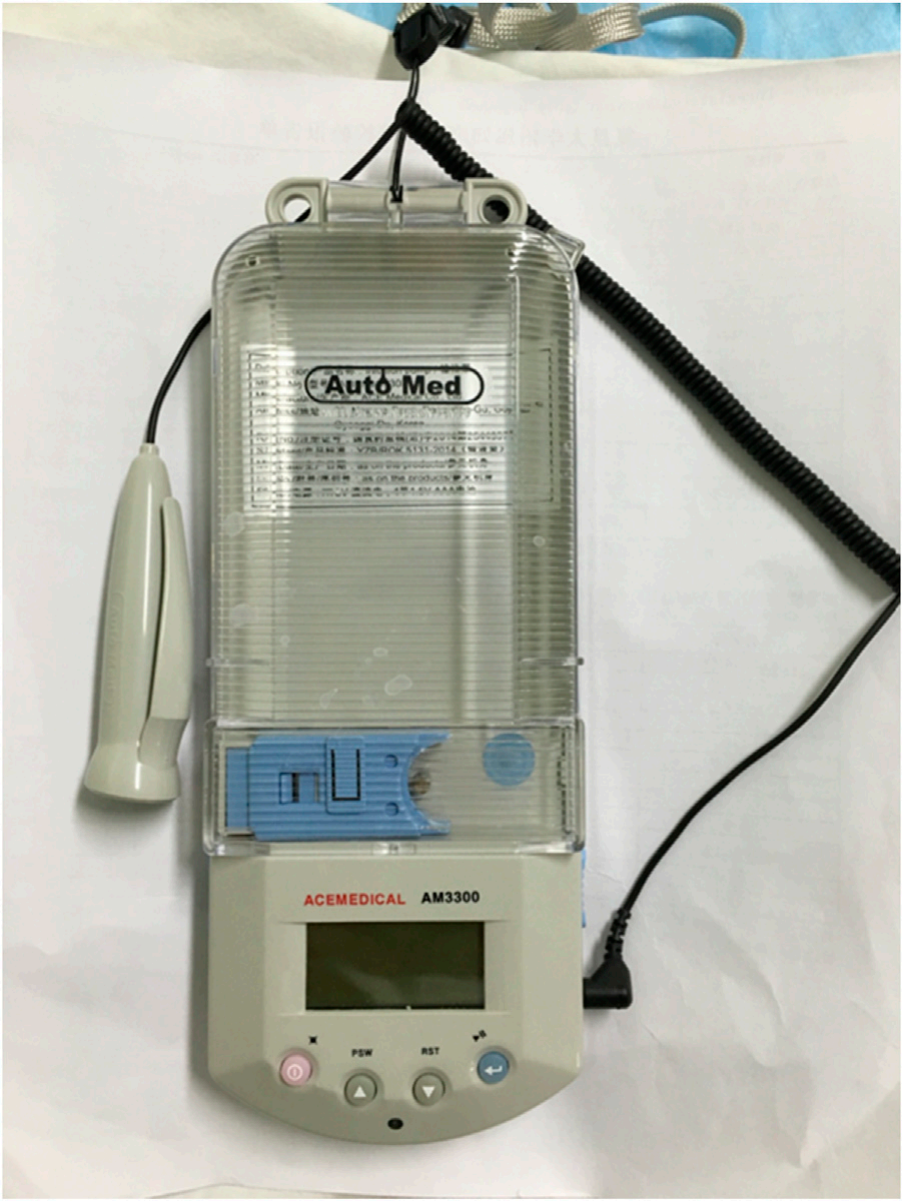
Patient-controlled intravenous analgesia pump picture.

Flurbiprofen axetil (50 mg) was intravenously administered to all patients at 0 and 6 h postoperatively. We instructed patients on the use of the PCIA pump (AM330, ACE Medical, Gyeonggi-Do, Korea) in the postoperative acute care unit (Figure 1). Patients complaining of severe pain postoperatively [a visual analog score (VAS) >5], were told to press the pump button to receive the bolus dose instead of receiving other rescue analgesics.

### Outcomes

The primary outcome of this study was pain determined by the VAS, total PCIA drug consumption, and the patient’s degree of satisfaction. The secondary outcomes included the sedation scores, lochia volume, time to initiation of lactation, and adverse events.

The same blinded physician (SS) explained the VAS to all patients to minimize subjective variations. A score of 0 indicated no pain and 10 indicated the worst possible pain. At each follow-up time-interval, detailed instructions explaining how to assess the VAS were read aloud and the patients then informed the same physician of the VAS score that best reflected their pain status. Patients were asked to report VAS of incision pain at rest (VAS-R) while shifting into a seated position (VAS-S), and of uterine cramping (VAS-U). VAS scores and total PCIA drug consumption were recorded at 6, 12, and 24 h after the operation.

The patient’s degree of satisfaction was assessed at 24 h on a scale of 0–3 (3, highly satisfied; 2, moderately satisfied; 1, somewhat satisfied; and 0, not satisfied). We used the Ramsay sedation scores (1, anxious patient; 2, cooperative and calm; 3, responding to commands; 4, brisk response to a stimulus; 5, sluggish response to a stimulus; 6, no response to stimulus) to assess sedation levels (Chiba et al., 2009). The amount of lochia was recorded for the initial 12 h after the operation by weighing sanitary napkins. We also recorded the time to initiation of lactation defined as the time from delivery to >10 ml of breast milk expressed through massaging both breasts. Patients were monitored for adverse reactions like hypotension (SBP <90 mmHg or DBP <60 mmHg), hypoxemia (SpO_2_ <90%), bradycardia (HR <60 bpm), respiratory depression (respiratory rate <10 breaths per minute for more than 10 min) and nausea and vomiting from the end of surgery until the termination of the PCIA. Hypotension or bradycardia was managed with phenylephrine or atropine, respectively, and respiratory depression was dealt with naloxone and oxygen.

### Sample Size Calculation

Under the supervision of the ethical committee a preliminary trial with 30 patients under combined spinal-epidural anesthesia was performed, in which the SD of VAS-U after the cesarean section was found to be 2. With a one-tailed *α* of 0.05 and power of 90%, to gain a difference of no less than 1.5 in VAS after cesarean section between two equal groups, a total of 38 patients in each group were required. To account for potential exclusions, a total of 84 patients (42 in each group) were enrolled in the study.

### Statistical Analysis

Graphpad Prism 5 (GraphPad Software, USA) and Stata 9.0 (StataCorp LP, College Station, Tex) were used for the statistical analysis. Data were presented as means ± SD and medians (interquartile ranges). The normalcy of data distribution was evaluated with normality plots and the Kolmogorov-Smirnov test. Numerical variables of normal distribution were compared using the student’s independent samples *t*-test. Ranked data were compared using the Cochrane-Mantel-Haenszel test. *p* < 0.05 was considered statistically significant.

## Results

**FIGURE 2:**
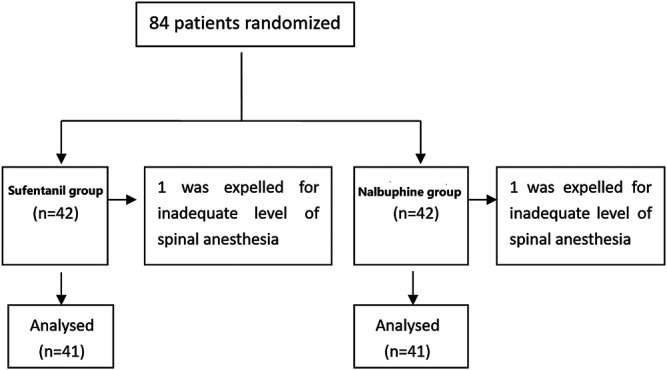
Study flow chart.

Eighty four patients were enrolled in the trial (42 in each group). Data of two patients (one from each group), who did not reach adequate levels of spinal anesthesia were excluded from the study (Figure 2). The characteristics of the included patients are presented in Table 1. Thirteen patients in the nalbuphine group and 11 in the sufentanil group received oxytocin or other uterine contraction agents. The total dose of PCIA per patient is reported in Supplementary Table S1. The mean 24 h doses in the nalbuphine and sufentanil group were 25.73 ± 0.51 mg and 27.02 ± 1.27 *µ*g respectively.

**TABLE 1:** Patient characteristics, lochia volume 12 h after delivery, and time to initiation of lactation in the two groups.

Variables	Sufentanil group(n = 41)	Nalbuphine group(n = 41)	*p* value
Age (years)	28.8 ± 6.0	28.6 ± 6.4	0.57
Weight (kg)	74.2 ± 9.2	73.9 ± 9.0	0.63
Height (cm)	161.8 ± 6.2	161.4 ± 6.3	0.68
Body mass index (kg/m^2^)	29.1 ± 7.4	29.0 ± 7.2	0.48
Duration of surgery (min)	53.2 ± 6.8	51.1 ± 6.3	0.36
Volume of lochia 12 h after delivery (ml)	97 (53)	101 (58)	0.76
Time to initiation of lactation (h)	54 (24)	56 (22)	0.77

Values are means ± SD or medians (interquartile ranges).

**FIGURE 3:**
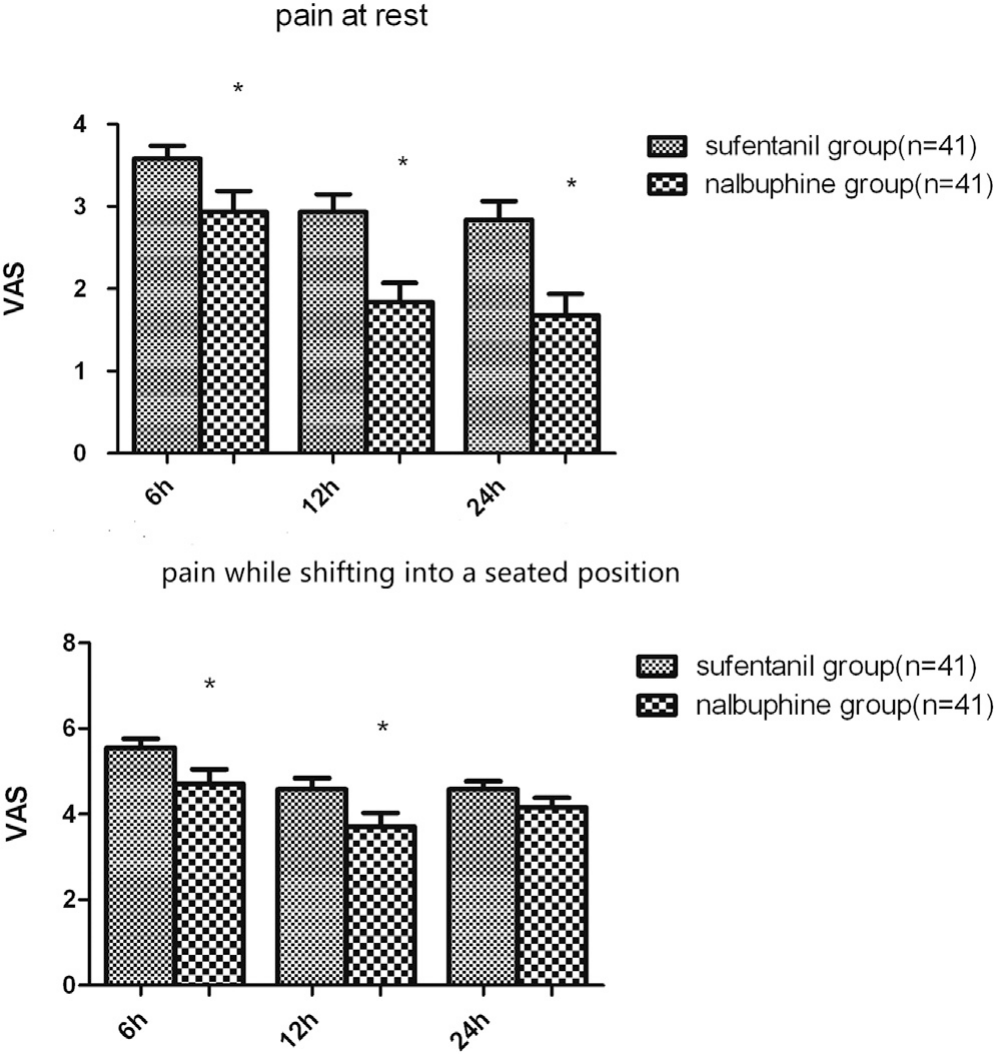
Comparison of mean postoperative pain at rest and while shifting into a seated position between the two groups. The mean visual analog score (VAS)-R in the nalbuphine group was significantly lower than that in the sufentanil group at three time points. Also, the mean VAS-S in the nalbuphine group was significantly lower than that in the sufentanil group at 6 and 12 h. Data are expressed as mean ± SD. ^*^p < 0.05 compared with sufentanil group.

**FIGURE 4:**
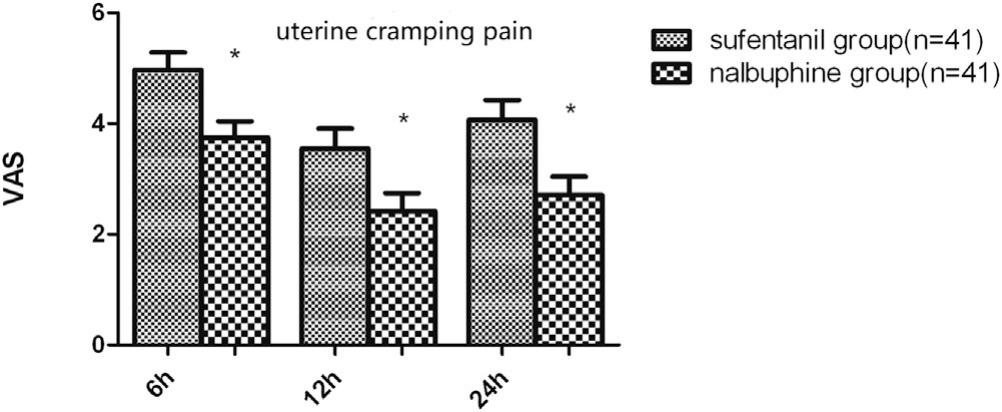
Difference in uterine cramping pain between the two groups. The mean visual analog score-U in the nalbuphine group was significantly lower than that in the sufentanil group at the three time points. Data are expressed as mean ± SD. ^*^p < 0.05 compared with sufentanil group.

**FIGURE 5:**
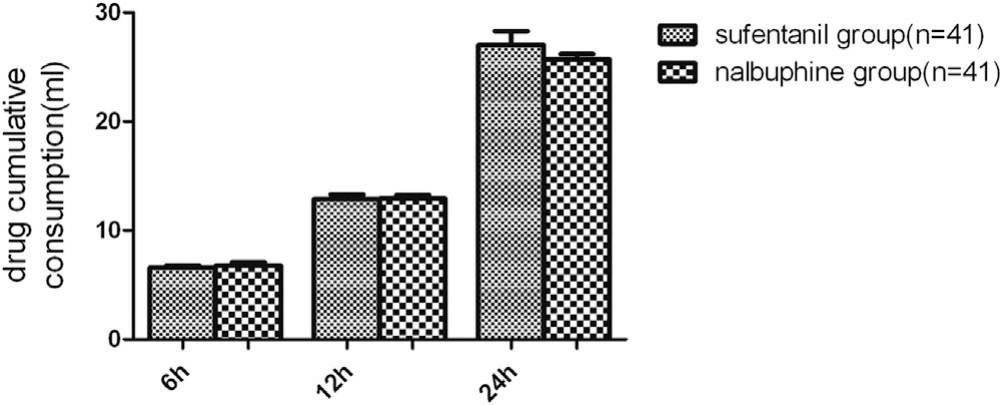
Comparison in cumulative drug consumption between the two groups. We found no significant differences in terms of the drug consumptions for patient-controlled intravenous analgesia between the two groups. Data are expressed as mean ± SD.

**FIGURE 6:**
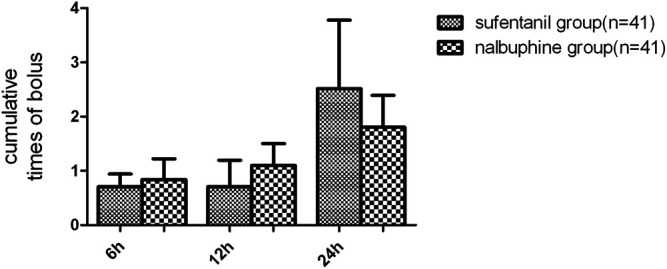
Comparison of cumulative patient-controlled intravenous analgesia (PCIA) bolus times between the two groups. Data are expressed as mean (SD). We found no significant differences in terms of the PCIA bolus times between the groups. Data are expressed as mean ± SD.

The mean VAS-R in the nalbuphine group at 6, 12, and 24 h (2.94 ± 0.25, 1.84 ± 0.23, 1.68 ± 0.26, respectively) were significantly lower than those in the sufentanil group (3.58 ± 0.16, 2.94 ± 0.21, 2.84 ± 0.23; *p* = 0.03, 0.001, 0.001, respectively; Figure 3). Mean VAS-S in the nalbuphine group at 6 and 12 h (4.71 ± 0.34, 3.71 ± 0.32, respectively) were significantly lower than those in the sufentanil group (5.55 ± 0.22, 4.58 ± 0.26) at the same time points (*p* = 0.04, 0.04, respectively; Figure 3). The VAS-U in the nalbuphine group at 6, 12 and 24 h (3.74 ± 0.30, 2.42 ± 0.32, 2.71 ± 0.36, respectively) were significantly lower than those in the sufentanil group (4.97 ± 0.32, 3.55 ± 0.36, 4.07 ± 0.36) at the same time points (*p* = 0.01, 0.02, 0.01, respectively; Figure 4). We found no statistically significant difference in PCIA drug consumption (Figure 5) and PCIA bolus times between the two groups (Figure 6). The degree of satisfaction in the nalbuphine group was significantly higher than that in the sufentanil group (*p* = 0.01; Table 2).

**TABLE 2:** Summary of the degree of satisfaction between the two groups.

Satisfaction degree	Sufentanil group (n = 41)	Nalbuphine group (n = 41)	*p* value
3, highly satisfied	3	9	0.01
2, moderately satisfied	31	31	—
1, somewhat satisfied	5	1	—
0, not satisfied	2	0	—

The Ramsay sedation scores were 2 points for all patients in both groups. We found similar volumes of lochia after delivery and time to initiation of lactation between the two groups (Table 1). Two patients in the nalbuphine group and one in the sufentanil group experienced nausea and vomiting. We encountered no patients with hypotension, bradycardia, or pruritus in either group.

## Discussion

Our findings suggest that the analgesic effect and patient satisfaction with nalbuphine PCIA are better than those of sufentanil PCIA in patients undergoing cesarean section. We found no significant differences in terms of lochia volumes after delivery, time to initiation of lactation, or the incidence of adverse effects between the two groups.

The analgesic effect of nalbuphine is similar to that of morphine, (Chiba et al., 2009; Zeng et al., 2015), while the ratio of the analgesic potency of sufentanil and morphine is 1:1,000 (Sun et al., 2020). Thus, by indirect comparison, the analgesic potency of sufentanil to nalbuphine can be considered to be 1:1,000. Hence, sufentanil 1 *µ*g is considered to be equivalent to nalbuphine 1 mg. The interactions between µ opiates and nalbuphine are complex. At low doses, nalbuphine appears to potentiate the effects of *µ* opiates; but at large doses, it seems to become an antagonist of *µ* opiates (Loomis et al., 1989). Yeh et al. (2008) have confirmed that the analgesic effect of a 1:1 ratio of morphine:nalbuphine is superior to the other ratio groups (1:3 or 3:1) for PCIA after gynecologic operations. Also as 100 *µ*g of sufentanil is commonly used for PCIA, hence equivalent doses of nalbuphine (100 mg) and sufentanil (100 *μ*g) were evaluated and compared in our study (Freye and Levy, 2008; Robert et al., 2015). The onset and duration of analgesic action of nalbuphine are similar to those of morphine, moreover, nalbuphine has a better safety profile with a lower incidence of adverse reactions like pruritus and respiratory depression) (Zeng et al., 2015). Only a limited number of studies have tested the efficacy of nalbuphine in cesarean sections. Culebras et al. (2000) have reported that the use of intrathecal nalbuphine 0.8 mg for cesarean sections produces a similar analgesic effect and fewer adverse events as compared to intrathecal morphine. Bindra et al. (2018) have demonstrated that intrathecal nalbuphine 0.8 mg and fentanyl 20 *μ*g are effective adjuvants to bupivacaine for subarachnoid blocks, but nalbuphine provides prolonged analgesia and can be a suitable alternative to fentanyl in cesarean sections. The efficacy of intravenous nalbuphine after a cesarean section has been tested by Chen et al. (2014). In a randomized controlled trial, the authors found that nalbuphine not only reduced the intrathecal morphine-induced pruritis but also significantly reduced total opioid consumption. While these past studies have differed in the routes of the drug administration, all have reported good analgesic efficacy of nalbuphine without any increase in the incidence of adverse events. Unlike previous studies, we segregated our pain scores into VAS-R, VAS-S, and VAS-U to better elucidate the difference of analgesic modalities on surgical pain and uterine cramp pain. It is known that the effect of analgesic drugs on visceral pain may be related to the κ receptor agonism (Riviere, 2004). This may be the reason for the significantly lower VAS-U scores in the nalbuphine group of our study. Further, our findings suggest nalbuphine provides better analgesia as compared to sufentanil for pain at rest as well as at seating. This may be important for patients with repeat cesarean sections because scar hyperalgesia may significantly add to the postoperative pain in them (Ortner et al., 2013). Our results concur with the study of Xi et al. (2020) who have reported reduced VAS scores with nalbuphine vs sufentanil in patients undergoing facial surgery. Similarly, Sun et al. (2020), have reported that patients on nalbuphine PCIA required a lesser dose of the drug as compared to sufentanil for pain control after a total hysterectomy. Since visceral pain is an important component of cesarean sections and hysterectomy procedures, and the fact that κ opioids are generally less potent in women (Lomas et al., 2007), the results of our study supplemented by previous literature suggest that use of nalbuphine may be a more rational and effective analgesic approach in women undergoing cesarean sections. Moreover, our findings of improved maternal satisfaction with nalbuphine in PCIA suggest the drug may be superior to sufentanil in such cases.

In our study, intravenous administration of nalbuphine did not lead to adverse effects such as hypotension, bradycardia, or pruritus. Only two patients had nausea and vomiting, and the safety profile of nalbuphine was found to be similar to that of sufentanil. Yeh et al. in their study have revealed that nalbuphine could decrease the incidence of morphine-induced pruritus in a dose-dependent manner (Yeh et al., 2008), Absence of pruritis in our study could be due to the low incidence of pruritus caused by sufentanil.

Jacqz-Aigrain et al. have reported that the relative infant dose of nalbuphine via intake of breast milk of the mother is 0.59 ± 0.27% of the weight-adjusted maternal daily dose (Jacqz-Aigrain et al., 2007). Thus, breastfeeding can be permitted after the administration of nalbuphine to the mother for postpartum pain. In our study, the mean 24 h nalbuphine dose to obtain satisfactory analgesia using PCIA was 25.73 mg. This was significantly lower than the 0.2 mg kg^−1^·4 h^−1^ dose (cumulative dose of 25.5 ± 34.5 mg/kg/ day) used in that study of Jacqz-Aigrain et al. (2007). However, it should be noted that Jacqz-Aigrain et al. (2007) did not use a continuous infusion of nalbuphine like our trial. Based on the elimination half-life of nalbuphine (t1/2 = 1.9 h) (Jaillon et al., 1989), the drug would have been washed out quickly when used as a bolus every 4 h. Thus, in our study nalbuphine was likely in a steady-state concentration in all the patients but at much lower levels. The mean time to initiation of lactation in our sample was between 54 and 56 h. Since nalbuphine infusions ended at 24 h after the cesarean section, based on the short t1/2, the expected concentration of nalbuphine in breast milk in our study would be extremely low, hence posts no risk to infants. Moreover, we found similar time to initiation of lactation in both groups, indicating similar effects on the time to initiation of lactation for both drugs.

We are aware of the limitations of our study. First, our results may only apply to women undergoing elective cesarean section after an otherwise uneventful pregnancy. Second, the study was not powered to assess the safety of the patients and neonates.

In conclusion, our study indicates that the administration of nalbuphine for PCIA after cesarean sections can provide improved analgesic effects with a higher degree of patient satisfaction as compared with sufentanil. The incidence of adverse events is not increased with the use of nalbuphine as compared to sufentanil. Further studies with a larger sample size are needed to confirm the results of our trials and to further evaluate maternal and neonatal safety of nalbuphine.

## Data Availability Statement

The raw data supporting the conclusions of this article will be made available by the authors, without undue reservation.

## Ethics-Statement

The ethics committee of Obstetrics and Gynecology Hospital of Fudan University approved this study with the approval number: 2017-22.

## Author Contributions

SS and TW: Data analysis, drafting of the paper. YG: Patient enrollment, data collection. SH: Study design.

## Funding

Youth Foundation from Shanghai Municipal Health Bureau (201344196).

## Conflict of Interest

The authors declare that the research was conducted in the absence of any commercial or financial relationships that could be construed as a potential conflict of interest.
