# Supplementary material for: Analgesic Effect Comparison Between Nalbuphine and Sufentanil for Patient-Controlled Intravenous Analgesia After Cesarean Section
Source: Front Pharmacol. 2020 Nov 16;11:574493. doi: 10.3389/fphar.2020.574493 (PMC7751695; doi:10.3389/fphar.2020.574493)
Supplement: Supplementary file 1 [file table1.docx]

Supplementary table S1: 24 hour drug consumption for each patient in the trial

| Patient No | Sufentanil group (μg) | Nalbuphine group (mg) | *P* value |
| --- | --- | --- | --- |
| 1 | 25 | 24 |  |
| 2 | 27 | 24 |  |
| 3 | 24 | 24 |  |
| 4 | 25 | 24 |  |
| 5 | 24 | 29 |  |
| 6 | 27 | 26 |  |
| 7 | 24 | 28 |  |
| 8 | 61 | 29 |  |
| 9 | 24 | 26 |  |
| 10 | 56 | 24 |  |
| 11 | 24 | 24 |  |
| 12 | 25 | 24 |  |
| 13 | 24 | 33 |  |
| 14 | 24 | 24 |  |
| 15 | 24 | 24 |  |
| 16 | 25 | 24 |  |
| 17 | 24 | 34 |  |
| 18 | 24 | 33 |  |
| 19 | 24 | 24 |  |
| 20 | 24 | 25 |  |
| 21 | 24 | 24 |  |
| 22 | 24 | 24 |  |
| 23 | 25 | 25 |  |
| 24 | 25 | 24 |  |
| 25 | 24 | 24 |  |
| 26 | 24 | 27 |  |
| 27 | 25 | 25 |  |
| 28 | 24 | 24 |  |
| 29 | 24 | 24 |  |
| 30 | 24 | 24 |  |
| 31 | 25 | 24 |  |
| 32 | 45 | 24 |  |
| 33 | 24 | 30 |  |
| 34 | 24 | 24 |  |
| 35 | 25 | 24 |  |
| 36 | 32 | 24 |  |
| 37 | 34 | 24 |  |
| 38 | 24 | 24 |  |
| 39 | 24 | 24 |  |
| 40 | 25 | 24 |  |
| 41 | 24 | 37 |  |
| Mean dose ±SD | 27.02 ± 1.27 | 25.73 ± 0.51 | 0.35 |

SD, standard deviation
